# *Salmonella* Infection in Pigs: Disease, Prevalence, and a Link between Swine and Human Health

**DOI:** 10.3390/pathogens12101267

**Published:** 2023-10-21

**Authors:** Laura Soliani, Gianluca Rugna, Alice Prosperi, Chiara Chiapponi, Andrea Luppi

**Affiliations:** Istituto Zooprofilattico Sperimentale della Lombardia e dell’Emilia-Romagna (IZSLER), 25124 Brescia, Italy; gianluca.rugna@izsler.it (G.R.); alice.prosperi@izsler.it (A.P.); chiara.chiapponi@izsler.it (C.C.); andrea.luppi@izsler.it (A.L.)

**Keywords:** *Salmonella*, swine, *S*. Choleraesuis, *S*. Typhimurium, *S*. 1,4,[5],12:i:-, *S*. Derby, pig meat, pig production chain, foodborne zoonoses, surveillance

## Abstract

*Salmonella* is one of the most spread foodborne pathogens worldwide, and *Salmonella* infections in humans still represent a global health burden. The main source of *Salmonella* infections in humans is represented by contaminated animal-derived foodstuffs, with pork products being one of the most important players. *Salmonella* infection in swine is critical not only because it is one of the main causes of economic losses in the pork industry, but also because pigs can be infected by several *Salmonella* serovars, potentially contaminating the pig meat production chain and thus posing a significant threat to public health globally. As of now, in Europe and in the United States, swine-related *Salmonella* serovars, e.g., *Salmonella* Typhimurium and its monophasic variant *Salmonella enterica* subsp. *enterica* 1,4,[5],12:i:-, are also frequently associated with human salmonellosis cases. Moreover, multiple outbreaks have been reported in the last few decades which were triggered by the consumption of *Salmonella*-contaminated pig meat. Throughout the years, changes and evolution across the pork industry may have acted as triggers for new issues and obstacles hindering *Salmonella* control along the food chain. Gathered evidence reinforces the importance of coordinating control measures and harmonizing monitoring programs for the efficient control of *Salmonella* in swine. This is necessary in order to manage outbreaks of clinical disease in pigs and also to protect pork consumers by controlling *Salmonella* subclinical carriage and shedding. This review provides an update on *Salmonella* infection in pigs, with insights on *Salmonella* ecology, focusing mainly on *Salmonella* Choleraesuis, *S*. Typhimurium, and *S*. 1,4,[5],12:i:-, and their correlation to human salmonellosis cases. An update on surveillance methods for epidemiological purposes of *Salmonella* infection in pigs and humans, in a “One Health” approach, will also be reported.

## 1. Introduction

*Salmonella* is one of the main intestinal pathogens in swine [1], and the infection in pigs is a cause for concern for two main reasons. The first is the threat that *Salmonella*-contaminated pork products pose to human health. The second is about animal health, with regard to clinical disease in pigs; mainly, septicemic salmonellosis associated with *Salmonella* Choleraesuis and enterocolitis associated with *S*. Typhimurium and its monophasic variant (*S*. 1,4,[5],12:i:-), which cause significant economic losses due to increased mortality, growth retardation [2,3,4], and cost of treatment [5].

Non-typhoidal *Salmonella* (NTS), together with *Campylobacter* spp., is one of the leading causes of foodborne disease at a global level [6], and the primary reservoir of NTS is the intestinal tract of warm-blooded animals, specifically livestock animals destined for food production, with the potential to lead to contaminated food products [7,8]. Eating contaminated food, especially foodstuffs of animal origin, is believed to be the major transmission source of *Salmonella* infections to humans, with a high burden on health systems internationally [7,9]. Human salmonellosis is mainly attributed to contaminated food consumption, such as poultry, eggs, pork, and beef, as well as fresh products [10]. Pork products are one of the main animal-derived foodstuffs involved in *Salmonella* transmission to humans [11,12]. It has been estimated that, every year, around 80.3 million human Salmonellosis cases have a foodborne origin [9]. In the European Union (EU), *Salmonella* is the second-most common source of foodborne infections in humans after *Campylobacter* since 2005 [13,14]. In the United States (US), according to the Centers for Disease Control and Prevention (CDC), illnesses caused by *Salmonella* are estimated to be around 1.35 million, with 26,500 hospitalizations and 420 deaths each year, with contaminated food acting as the main source of infections [15]. It is also estimated that foodborne salmonellosis costs the US $2.7 billion per year, with pork producers losing approximately $100 million [16]. According to recent data, as of January 2020, there were approximately 677.6 million pigs worldwide [17]. The pig production system has undergone great changes in the past century, transitioning from small herds to large holding facilities housing a great number of animals [5,18]. The exponential growth of intensive farming and food production practices could have led to new issues regarding the management and control of swine salmonellosis [11,19]. Moreover, available data on the incidence of *Salmonella* infection in pigs are scarce and often represent only the tip of the iceberg, mainly because pigs infected with *Salmonella* are often subclinical carriers [20]. Given that the pig production chain and, consequently, pig meat consumption have a direct impact on human health, a more collaborative approach between human and veterinary medicine (“One Health”) is crucial for preventing and minimizing potential health threats along the food chain, in order to preserve animal, human, and environmental safety [21]. 

The purpose of this review is to update the information on clinically relevant *Salmonella* serovars (*S*. Choleraesuis, *S*. Typhimurium, and its monophasic variant) responsible for infection and disease in pigs. *Salmonella* Derby will also be discussed, given its strong association with the pig production chain, even if it is not considered a cause of enteric disease in pigs. Moreover, the impact of these serovars on human health will also be evaluated from a “One Health” point of view.

## 2. Etiology

The genus *Salmonella* is divided into two species: *S*. *enterica* and *S*. *bongori*. Moreover, the species *enterica* is divided into six different subspecies: *enterica* (I), *salamae* (II), *arizonae* (IIIa), *diarizonae* (IIIb), *houtenae* (IV), and *indica* (VI). There are more than 2600 different *Salmonella* serovars, each characterized by a distinct antigenic formula [22,23]. While *S*. *bongori* is mainly isolated from cold-blooded animals and from environmental sources, *S*. *enterica* subspecies *enterica’s* main reservoir are warm-blooded animals, which harbor the pathogen in their intestinal tract [24,25]. Moreover, bacteria belonging to the genus *Salmonella* are well known for their capability to infect an ample range of hosts, often exhibiting different behaviors regarding their host range [26]. The majority of NTS, namely any serovar different from *Salmonella* Typhi or *Salmonella* Paratyphi A, B, and C [27], are characterized by having a broad host range, and are otherwise called “generalist” serovars, e.g., *S*. Typhimurium, which can infect and cause disease in several animal species, including humans, pigs, cattle, and poultry [7]. Nonetheless, there are serovars which are adapted or restricted to a single host species; for example, *Salmonella* Dublin is adapted to cattle [28], *Salmonella* Gallinarum is restricted to fowl [29], and *S*. Choleraesuis is adapted to pigs [30]. 

## 3. *Salmonellosis* in Swine

In swine, clinical salmonellosis has been mostly associated with two serovars: *S*. Choleraesuis, especially the Kunzendorf variant, and *S*. Typhimurium [30]. Clinical disease is usually characterized by systemic disease with septicemia (often associated with pneumonia), mainly caused by the *S*. Choleraesuis serovar; and enteric disease, characterized by diarrhea, which is typically due to *S*. Typhimurium and *S*. 1,4,[5],12:i:- [30,31]. Even though not associated with enteric disease in pigs, *S*. Derby will also be discussed, given its relevance to the pig production chain. 

### 3.1. S. Choleraesuis

#### 3.1.1. Overview

The first report of *S*. Choleraesuis dates back to 1886 in the US, when it was isolated by Salmon and his assistant Smith, who believed it to be the causative agent of “hog cholera” (swine fever) [32]. From a serological point of view, *S*. Choleraesuis shares its antigenic formula (6,7:c:1,5) with the *S*. Paratyphi C and *Salmonella* Typhisuis serovars. In addition, three *S*. Choleraesuis biotypes have been identified: *S*. Choleraesuis *sensu stricto*, *S*. Choleraesuis variant Kunzendorf, and *S*. Choleraesuis variant Decatur; these variants share the same antigenic formula but have different biochemical characteristics (dulcitol and mucate fermentation, H_2_S production) [22].

*S*. Choleraesuis is considered a swine-adapted serovar, but it is not host-restricted, given that it is also capable of causing invasive disease in humans [33,34,35,36,37], and while it is rarely reported in Europe, it is still frequent in Asia [13,35,37,38].

Historically, most *S*. Choleraesuis outbreaks in swine, characterized by septicemic forms, have been caused by the H_2_S-producing Kunzendorf variant [30,31,39].

In the US, *S*. Choleraesuis was the predominant serovar isolated from swine in the 1950s and the 1960s [31]. This predominance continued up until the mid-1990s. After 1995, there was a shift in the main serovars isolated from swine in the US, with a decrease in *S*. Choleraesuis prevalence and an increase in reported isolations of *S*. Typhimurium, *S*. 1,4,[5],12:i:-, and *S*. Derby, which are now the predominant serovars in swine [10].

In the EU, as of now, *S*. Choleraesuis is not widespread in pigs anymore [40], as it is isolated at low frequency in some European countries (e.g., Estonia and Romania) [13,38]. Data obtained by the 2006–2007 EFSA baseline survey, which aimed to evaluate *Salmonella* prevalence in slaughter pigs, highlighted that *S*. Choleraesuis was reported in only 4 out of 25 Member States (MSs, namely any nation that is a member of the European Union), with only 10 positive samples out of 2600 serotyped ileocecal lymph nodes [41]. Later on, *S*. Choleraesuis variant Kunzendorf accounted for 2.5% of all serotyped *Salmonella* isolates from pigs in the EU in 2013 [14]. According to the latest EFSA-ECDC report on zoonoses, out of all serotyped *Salmonella* isolates in pigs (from both animal and food sources), *S*. Choleraesuis *sensu stricto* and variant Kunzendorf were reported only six times each, with a very low prevalence of 0.38% [13]. Nowadays, in the EU, there are still reports about the isolation of *S*. Choleraesuis from pigs in countries such as Italy [42] and Serbia [43]. With regard to Northern Europe, after an absence of 13 years, *S*. Choleraesuis var. Kunzendorf was detected for the first time in swine herds in Denmark between 2012 and 2013, with reported outbreaks characterized by high mortality rates (20–30%) [39]. Moreover, after 40 years of absence, *S*. Choleraesuis was reported in 5 pig herds in Sweden in 2022 [44].

Furthermore, it is worth mentioning that wild boars are considered a natural wildlife reservoir for *S*. Choleraesuis, given that this serovar has been isolated multiple times from this species, with the potential for generating spillover events between wildlife and farmed animals [32,44,45]. In the EU, there has been a growing trend of *S*. Choleraesuis isolation from wild boars, whose number has been increasing in the past few decades [46]. Various outbreaks caused by *S*. Choleraesuis var. Kunzendorf have been reported in the wild boar population [47,48], as well as its isolation from sickened and killed wild boars, characterized by septicemic salmonellosis [49,50]. In Sweden, it was reported that, out of a total of 633 wild boars sampled within surveillance programs, 80 animals tested positive for *S*. Choleraesuis var. Kunzendorf, and all sequenced isolates clustered with those isolated from domestic pig farms in the same region [44]. This highlights the importance of implementing strict biosecurity measures to prevent and hinder the possibility of transmission events from the wild boar population to domestic pigs [50].

#### 3.1.2. Clinical Signs and Gross Lesions

**Figure 1:**
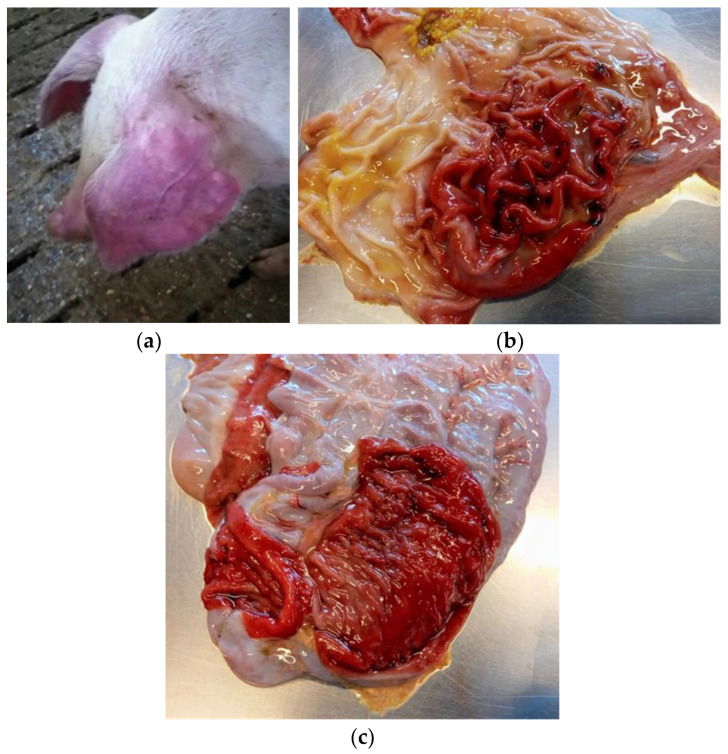
(**a**) Pig affected by salmonellosis (*S*. Choleraesuis). Cyanosis, with obvious red-blue discoloration of the ears. (**b**) Pig affected by salmonellosis (*S*. Choleraesuis). Severe gastritis and ulcers are observed in the gastric mucosa. (**c**) Pig affected by salmonellosis (*S*. Choleraesuis). Severe colitis with particularly reddened colon mucosa.

In pigs infected with *S*. Choleraesuis, typical signs of acute disease are cyanosis and dyspnea, with red-blue discoloration affecting mainly the ears, the chest, and the abdominal area, but also feet, belly, and ventral neck, sometimes associated with enterocolitis [31,43,51,52] (Figure 1a).

Among other common clinical manifestations, there are pyrexia, inappetence, drowsiness, moist cough, and labored breathing, while diarrhea is less pronounced or absent. Usually, clinical signs tend to arise 24–36 hours after infection, while gastrointestinal signs typically appear after 4–5 days [31]. During most outbreaks, morbidity tends to be variable, while mortality is generally high [31].

Typical lesions induced by *S*. Choleraesuis infection in swine are splenomegaly, gastritis, gastric mucosa infarction and gastric erosions (Figure 1b), swollen and enlarged mesenteric and gastro-hepatic lymph nodes, lung congestion and pneumonia, random white foci of necrosis in the liver, and enterocolitis (Figure 1c) [30,31].

The main microscopic lesions are characterized by scattered foci of coagulative necrosis with neutrophils and histiocytes infiltrates in the liver. Similar lesions can be detected in the spleen and in lymph nodes. Fibrinoid thrombi can be observed in the venules of cyanotic skin, gastric mucosa, kidneys (affecting glomerular capillaries), and pulmonary vessels [30]. The lungs can show interstitial pneumonia or suppurative bronchopneumonia. Segmental necrotizing vasculitis with perivascular histiocytic infiltrates, sometimes with localized necrotizing encephalitis, is uncommonly observed [30].

### 3.2. S. Typhimurium and Its Monophasic Variant S. 1,4,[5],12:i:-

#### 3.2.1. Overview

Even though a broad range of *Salmonella* serovars can infect pigs, only a restricted number of serovars are considered a primary source of disease, with *S*. Typhimurium and *S*. 1,4,[5],12:i:- being among the most important ones [53]. *S*. 1,4,[5],12:i:- is a relatively recent serovar, having been isolated from poultry in the late 1980s in Portugal [54]. *S*. 1,4,[5],12:i:- is characterized by the lack of expression of the phase 2 flagellar antigen (H2), encoded by locus *fljB* [55]. This could be due to different mutations (point mutations included) or to complete or partial deletion in the *fljB* locus or in neighboring genes [56]. According to the White–Kaufmann–Le Minor scheme, when it was first isolated, the strain could either be a monophasic variant, *S*. Typhimurium, *S*. Lagos, or an uncharacterized serotype [22,57]. Further investigations, which involved methods such as pulsed-field gel electrophoresis (PFGE), phage typing, plasmid profiling, and multilocus VNTR analysis (MLVA), allowed researchers to determine that there was indeed a close genetic relatedness between *S*. 1,4,[5],12:i:- and *S*. Typhimurium [57,58,59]. Therefore, *S*. 1,4,[5],12:i:- is now widely accepted as a *S*. Typhimurium variant [60]. After its discovery, *S*. 1,4,[5],12:i:- has become one of the most widespread *Salmonella* serovars worldwide and one of the most reported serovars from human salmonellosis cases [56,60,61]. Probably, *S*. 1,4,[5],12:i:- possesses competitive and evolutionary advantages compared to other biphasic strains, and this has contributed to its increasing predominance worldwide; nevertheless, further studies are needed to investigate the reasons that allow the successful spread of monophasic strains [53,62,63,64].

An Italian study reported data about the isolation of *S*. Typhimurium and *S.* 1,4,[5],12:i:- in 1359 pig farms in Northern Italy, where clinical enteric forms or mortality occurred. *S*. Typhimurium and *S.* 1,4,[5],12:i:- represented 12.23% and 6.18% of the isolated serovars, respectively. In this study, the association between the isolated serovars and the presence of clinical signs showed a stronger correlation with *S*. Typhimurium compared to *S.* 1,4,[5],12:i:- [65].

It was suggested that, probably, the different antigenicity and pathogenicity of *S.* 1,4,[5],12:i:- make the infection harder to recognize and control [65]. Furthermore, when compared to *S*. Typhimurium, a number of factors (including prophage involvement and antigenic changes) can cause a reduced immune response in pigs to *S*. 1,4,[5],12:i:- [62].

In an experimental challenge study, three groups of piglets were inoculated with *S*. Typhimurium, *S*. Derby, and *S*. 1,4,[5],12:i:-. Despite the fact that all groups displayed diarrhea, no fever was detected in the group challenged with *S*. 1,4,[5],12:i:-, and only piglets challenged with *S*. 1,4,[5],12:i:- shed *Salmonella* continuously throughout the trial, with higher excretion levels than the group challenged with *S.* Typhimurium [66]. These data allow for hypothesizing a competitive and selective advantage of *S.* 1,4,[5],12:i:- over *S*. Typhimurium, which may be aided by microevolution affecting antigenicity, pathogenicity, and transmission [67]. Because of its high transmission ability, this serovar has become a global public and animal health hazard [56].

#### 3.2.2. Clinical Signs and Gross Lesions

**Figure 2:**
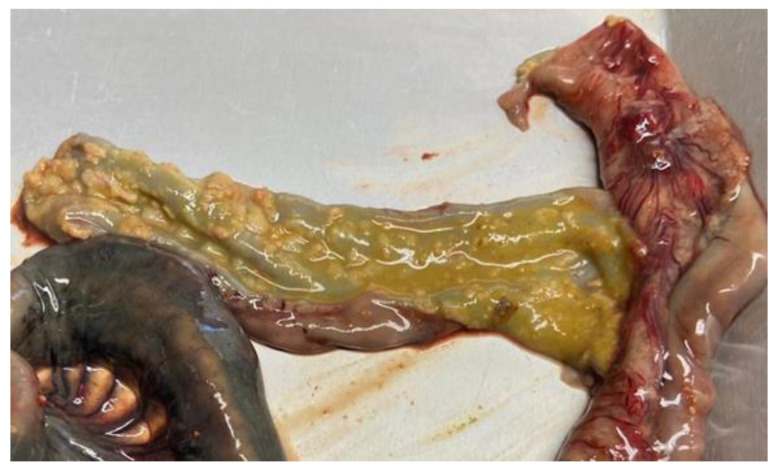
Pig affected by salmonellosis (*S*. Typhimurium). Necrotic enterotyphlocolitis with diphtheritic membrane on the mucosal surface.

*Salmonella* infections in pigs are frequently asymptomatic, even though fecal shedding can occur continuously or intermittently, even over long periods of time, regardless of the presence of clinical signs. In symptomatic pigs, indistinguishable clinical signs can be observed after infection with *S.* Typhimurium and *S*. 1,4,[5],12:i:- [68], which are characterized by yellow diarrhea (rarely containing blood), dehydration, decreased feed intake, fever, inanition, and wasting. Debilitating conditions, such as poor hygiene and viral infections that induce immunosuppression, e.g., porcine reproductive and respiratory virus (PRRSV) and porcine circovirus 2 (PCV2), can trigger the onset of disease. Mortality is usually low and due to hypokalemia and dehydration, often occurring after several days of diarrhea [68]. After complete clinical recovery, a percentage of pigs can act as carriers and intermittent shedders for at least 5 months. The persistence of the organism is particularly frequent in submandibular and ileocolic lymph nodes, tonsils, and in the large intestine [68]; in addition to that, stressful conditions for the animals, e.g., commingling, transport, diet change, and lairage can increase shedding [69,70]. A few pigs may remain chronically wasted, and some may show obstipation and marked distension of the abdomen. This condition is described as a consequence of rectal strictures due to defective healing, with fibrosis, of ulcerative proctitis caused by *S.* Typhimurium [30]. Necrotic enterotyphlocolitis is the most consistent gross lesion in pigs suffering from *S*. Typhimurium. The lesions are mainly recorded in the ileum, cecum, and spiral colon, with the formation of diphtheritic membranes on the mucosal surface and roughened mucosa with a granular appearance. Systemic dissemination and septicemia are rare [68] (Figure 2).

Multifocal or coalescing mucosal erosions and ulcers may be detected, associated with adherent grey-yellow fibrino-necrotic material, while mesenteric lymph nodes are markedly enlarged and congested [30].

### 3.3. S. Derby

As opposed to *S*. Choleraesuis and *S*. Typhimurium, *S*. Derby is not known to be a cause of significant enteric disease in pigs, despite being frequently isolated from swine [53]. During a study in which pigs were challenged with *S*. Typhimurium, *S*. 1,4,[5],12:i:-, and *S*. Derby, no visible gross lesions were observed in necropsied pigs infected with *S*. Derby, as opposed to those infected with *S*. Typhimurium and *S*. 1,4,[5],12:i:-. With regard to histological lesions (mainly in the cecum and spiral colon), higher scores were registered for piglets infected with *S*. Typhimurium and *S*. 1,4,[5],12:i:- compared to those challenged with *S*. Derby [53]. Given this, *S*. Derby is known for its ability to cause durable asymptomatic infections in swine [71]. However, in another study in which piglets were inoculated with *S*. Derby, the animals showed diarrhea and fever [66]. As a matter of fact, it has been demonstrated that piglets experimentally challenged with *S*. Derby shed the pathogen in the feces at higher levels compared to those challenged with *S*. Typhimurium [72]. Taken together, these data could explain the wide diffusion of this serovar in pigs and why *S*. Derby is among the most reported serovars linked to the pork industry [73].

## 4. *Salmonella* Choleraesuis, *S*. Typhimurium, and *S*. 1,4,[5],12:i:- Infection in Swine: Carrier State and Environmental Persistence

Among the main *Salmonella* transmission sources on farm grounds, there are the introduction of infected animals [74], contaminated feed [75,76,77], and pests, such as rodents, birds, and flies, which can act as *Salmonella* vectors [31,78,79]. However, asymptomatic *Salmonella* carriers are one of the main infection sources, with the fecal–oral route being the main way of *Salmonella* transmission among pigs [76]. There has also been evidence of the role played by the upper respiratory tract as an equally important route of *Salmonella* infection in swine [80].

### 4.1. S. Choleraesuis

A study involving experimental infection in pigs with *S*. Choleraesuis evidenced that the pathogen was prevalently recovered from ileocolic lymph nodes, tonsils, lungs, colon, and cecal content [81]. Following oral transmission, tonsils become rapidly contaminated with *Salmonella*, which can then be found in oropharyngeal secretions; this could allow for nose-to-nose transmission [30]. In a study in which pigs were challenged intranasally with *S*. Choleraesuis, the bacterium could persist in the tonsils for up to 19 weeks post-infection. These data led researchers to hypothesize that tonsils could be a crucial site for *S*. Choleraesuis maintenance in swine herds [82]. In addition, *S*. Choleraesuis spread and persistence at herd level is strongly influenced by the carrier state recovered animals can acquire, shedding the bacteria through their feces for extended periods of time [51]. It was also demonstrated that, in pigs challenged with 10^8^ CFU/mL of *S*. Choleraesuis, the carrier state could be maintained for up to 12 weeks, regardless of the inoculation route [82]. An experimental infection of neonatal piglets with *S*. Choleraesuis was performed to evaluate the pattern of long-term shedding, which revealed continuous shedding for up to 85 days after infection [83]. Given that piglets infected with *S*. Choleraesuis rarely exhibit clinical signs, shedding by subclinically infected piglets could play a role in the spread of *Salmonella* on farm grounds [83]. In addition to that, the carrier state, as well as the frequency and duration of shedding, are not influenced by the use of antibiotics in pigs. This differs from what is described in human enteric salmonellosis, where the carrier state is prolonged by the use of antibiotics [84,85,86,87,88,89].

The main routes of *S.* Choleraesuis transmission in swine herds are believed to be the horizontal transfer from carrier or diseased pigs to healthy individuals [83], and the facilities which were previously contaminated by this serovar [77]. In contrast, it is rare to find *S*. Choleraesuis in feed or in animals other than pigs [30,31]. Additionally, it has been proven that *S*. Choleraesuis is capable of surviving in wet swine feces for 3 months after shedding, while in dry feces, the bacterium survives for at least 13 months. Hence, *S*. Cholerasuis is capable of remaining viable and infective in the environment for extended periods of time [90]. Furthermore, *S*. Choleraesuis can remain dormant in swine herds and then be activated by other diseases, such as coinfection with PRRSV or PCV2, which induce an immunosuppressed condition in affected pigs [48].

### 4.2. S. Typhimurium and S. 1,4,[5],12:i:-

After infection with *S*. Typhimurium and its monophasic variant, the majority of pigs recover completely, while a lower portion acquire the carrier state, shedding the pathogen intermittently for up to 5 months [30]. After challenge, *S.* Typhimurium has been shown to persist in low numbers in swine for up to 28 weeks; moreover, after oral experimental challenge in pigs, *S*. Typhimurium showed a marked tropism for the tonsils [74,81,82]. The highest excretion rates were recorded at 2 weeks post-infection in a study in which 42-day-old piglets were orally challenged with 10^9^ CFU of *S*. Typhimurium; after that, shedding decreased and became intermittent. Moreover, this study evidenced that long-term *S*. Typhimurium persistence in swine is restricted to the tonsils, the gastrointestinal tract, gut-associated lymphatic tissue, and mesenteric lymph nodes [91].

Another study evaluated *S*. Typhimurium prevalence on a pig farm, characterized by recurrent infections with this serovar. The authors detected the same *S*. Typhimurium PFGE profile from fecal samples isolated from farm grounds, waste slurry, agricultural soil spread with *Salmonella*-positive animal waste, and also from samples isolated from asymptomatic carrier pigs. These findings highlighted the ability of *S*. Typhimurium to persist in the environment, particularly in agricultural soil, for 14 days after spreading contaminated slurry. This persistence could fuel a cycle of continuous reinfection in herds. As a result, adequate and effective waste management practices are critical to contrast the long-term survival of *Salmonella* on farm grounds [92]. In addition, *S*. Typhimurium long-term survival in pig slurry was also reported (34 days in artificially contaminated slurry) [93]. It was also reported that *Salmonella* can survive for up to 50 months in the environment (e.g., slurry and dust) [94]. In addition to that, it has been demonstrated that *S*. Typhimurium can survive for up to 5 weeks in soil and up to 7 weeks in pig shelter huts, further leading to environmental contamination [95].

In another study, in which swine feces were artificially inoculated with both *S*. Typhimurium and *S*. 1,4,[5],12:i:-, bacteria were able to survive for 88 days [96]. Moreover, in a study in which pigs were exposed to low environmental doses of *S*. 1,4,[5],12:i:-, the excretion rate reached 10^4^ CFU/g in feces [97]. For *S*. Typhimurium, both low, persistent carriers, as well as super shedders, have been documented [16,98]. Taken together, these findings confirm that sick animals’ shedding of *Salmonella* can contaminate the environment, continuously fueling a cycle of reinfection in newly introduced animals [90].

## 5. Prevention and Control of *Salmonella* Infection in Pig Farms and at Slaughter

Humoral immunity to *Salmonella* infections has a limited effect because for much of the infection cycle, the organism stays within body cells, shielded from antibody action [99]. However, there is a strong humoral response to natural infection, including secretory IgA responses that may be effective in preventing the initial invasion of the mucosa [100]. Cell-mediated immunity (CMI), characterized by a T-helper1 (Th1) lymphokine profile associated with the activation of macrophages and cytotoxic lymphocytes, appears to be a critical part of effective anti-*Salmonella* immunity [101]. In general, for particular strains, such as host-adapted *S.* Choleraesuis, it is often necessary to control the outbreaks of clinical disease, which have an important economic impact in terms of mortality and antibiotic consumption. Vaccination can be an effective tool to control *Salmonella* infections at the farm level. This approach has substantial differences when the goal of vaccination is to protect the consumers of pig products by controlling subclinical carriage and shedding [99]. In this latter case, vaccination should guarantee:

1. The control of a broad variety of strains and serovars;
2. The reduction in tissue colonization and/or shedding at the time of slaughter;
3. No adverse effect on serological monitoring for *Salmonella* infection where this is employed before or at the time of slaughter, as performed in Denmark and other European countries to categorize the level of *Salmonella* infection in pig herds [102].

It has been demonstrated that vaccination against *S*. Choleraesuis cross-protected pigs against other strains, such as *S*. Typhimurium [103,104] and *S*. Derby [105], showing a certain degree of cross-protection between serovars [106]. Despite this, it is generally accepted that serovar-specific vaccines are more likely to be effective, as antibodies induced by different *Salmonella* serovars show only a low level of cross-protection [106].

In experimental challenge and field studies, inactivated vaccines with appropriate administration protocols and adjuvants showed protective effects against antigenically similar strains [99], whereas live vaccines should be able to cross-protect vaccinated animals against different *Salmonella* serogroups. The use of an autogenous, inactivated vaccine prepared from outbreak strains is a rapid intervention that may be effective, in concert with other control measures, if a licensed commercial vaccine is not available [107,108]. Alborali and colleagues (2017) showed that the combination of *S*. Typhimurium- and *S*. Choleraesuis-attenuated and inactivated vaccines, respectively, is effective against challenge infection with *S*. Choleraesuis. As, in field conditions, the simultaneous infection with more than one serovar is common, these data suggest that the development of a new effective vaccine with this strategy could be promising in tackling the effects of *Salmonella* infection [109].

Vaccination is widely accepted to play a role in reducing *Salmonella* prevalence in pigs, and it may become an adjunct to on-farm control [110], by preventing *Salmonella* from colonizing the gut and, as a result, reducing subsequent shedding and the development of the carrier state [111]. Moura and colleagues (2021) showed that vaccinating pigs twice (with an interval of 21 days) with a commercial inactivated vaccine containing strains of *S.* Choleraesuis, *Pasteurella multocida*, and *S.* Typhimurium, and then performing an oral challenge with 10^8^ CFU of *S.* Typhimurium, partially protected the animals, reducing *Salmonella* excretion in feces and the colonization of organs [112]. The strategy combining maternal and pre-weaned piglets’ vaccination is needed for better protection against challenge in the post-weaning period [99]. Nonetheless, vaccination alone is not sufficient to eliminate the infection; a combination of different strategies such as biosecurity measures, cleaning and disinfection procedures, and feeding practices is required in order to reduce *Salmonella* prevalence in pigs.

An all-in/all-out production system can prevent cross-contamination between production cycles by allowing thorough cleaning and disinfection and, consequently, reducing the potential of *Salmonella* exposure and infection in subsequent batches [113]. This is of huge importance, considering that *Salmonella* can persist in the environment for several months to years [92,94]; furthermore, the farm environment can act as a *Salmonella* reservoir because of inadequate disinfection. In addition, *Salmonella* intestinal infection is positively correlated with ambient temperature (between 35 °C and 37 °C), as warmer temperatures enable rapid replication [114].

To limit *Salmonella* persistence and spread in farms and lairage environments, appropriate cleaning and disinfection regimes must be implemented. A significant reduction in the prevalence of *Salmonella* in pigs in appropriately cleaned and disinfected buildings was demonstrated, as well as the correct use of boot dips [115]. As an example, Gradel and colleagues (2004) reported that the preferred class of disinfectants appears to be peroxygen-based products [116]. Other disinfectants effective against *Salmonella* are glutaraldehyde, quaternary ammonium compounds (QACs), iodine-based compounds, and chlorocresols [78]. Disinfectants’ effectiveness may be compromised by the presence of organic matter or by over-dilution if used before the surfaces are completely dried [116]. The drying of pens greatly reduces the probability of detecting *Salmonella*, and its complete elimination was reported to be achieved 24 hours after cleaning with detergents and a chlorocresol-based disinfectant [117]. Smooth surfaces are less likely to have a high level of residual contamination than rough ones [118]. This is important, especially because concrete is a material that is widely used in pig farrowing accommodations and its rough surface could harbor higher numbers of bacteria. It has been reported that *Salmonella* contamination is very quickly transferred to roads, standing water, transport trucks, and other mobile equipment [119], thus making vehicles’ disinfection a crucial part of any rigorous biosecurity practices [120]. Fecal contamination of feed, drinkers, or farm equipment by rodents, wild birds, insects, and pets (dogs and cats) can represent a possible way for the introduction and transmission of *Salmonella* to pigs [121,122]. In particular, rodents can efficiently contribute to spreading *Salmonella* as they are very efficient vectors and amplifiers of the pathogen [115]. Rodenticides and biosecurity procedures, as well as the disposal of dead animal and feed remains, should be used in combination for efficient pest control [122].

Furthermore, the feed form is a very important risk factor. Wet feed is preferred to dry feed [123], while dry meal feed is preferred over pelleted feed, as long as the feed particle size of the meal is not too small [124]. Given that pellets have to be made from very finely ground ingredients to maintain their integrity, this reduces the transit time through the digestive tract and therefore does not achieve a low protective intestinal pH. On the contrary, coarsely ground meal decreases the survival of *Salmonella* during stomach passage because of slower gastric transit and lower gastric pH [125], thus improving productivity in pigs and delaying exposure to *Salmonella* [126]. Heating treatment of feed, performed at 93 °C for 90 seconds with 15% moisture, may eliminate *Salmonella* [127], even if the contamination level and possible post-treatment contamination are critical factors.

Manure management is important in order to reduce the risk of introduction and spread of *Salmonella*, as well as other pigs’ infectious agents. For this reason, manure treatments should be implemented in order to reduce or kill *Salmonella*. Anaerobic digestion, composting, and separation technologies are used for this purpose [128].

All these mitigation strategies should be coupled with appropriate measures to reduce the risk of carcass contamination by *Salmonella* at slaughter [129]. Pigs entering the slaughter line are first stunned, killed, and then exsanguinated. After that, a series of treatments, e.g., scalding, dehairing, singeing, and polishing, are performed on carcasses in order to lower microbiological contamination [130]. All of these steps should be performed in accordance with good manufacturing practices (GMPs) and following strict cleaning protocols and hygiene practices [130]; for example, it has been demonstrated that scalding water should always be kept between 60–62 °C to keep the water free from *Salmonella* contamination [131]. The aforementioned stages are followed by evisceration, which is one of the crucial stages in the slaughtering process; the accidental leakage of intestinal content due to perforation is one of the leading causes of *Salmonella* carcass contamination [130]. Moreover, slaughterhouse personnel’s hygiene and utensil cleaning and sanitation are necessary to avoid permanent contamination [130]; for these reasons, personnel should be properly trained about correct working procedures. In addition, proper carcass splitting, decontamination, and chilling procedures should be performed to at least reduce, if not eliminate, bacterial contamination [131]. The control of transportation and refrigeration temperature is crucial in hindering *Salmonella* outgrowth during food storage at retail level [131]. Lastly, correct food handling practices are to be adopted, coupled with high hygienical standards and proper cooking temperatures, in order to avoid possible cross-contamination and to lower the risk of infection in humans [131].

## 6. *Salmonella* in Swine Farms and in the Pig Production Chain

### 6.1. Salmonella Prevalence in Swine Farms

The EFSA baseline survey conducted in 2008, in which pooled fecal samples were analyzed, reported an overall *Salmonella* prevalence of 31.8% and of 33.3% for pigs’ breeding and production holdings, respectively, in the EU. It was also reported that *S*. Typhimurium had a prevalence of 25.4% and of 20.1% in breeding and production holdings [132]. In more recent years, *S*. 1,4,[5],12:i:- and *S*. Typhimurium were among the most isolated serovars from pigs in the EU (Table 1) [13,133,134,135].

Furthermore, a retrospective analysis of *Salmonella* serovars isolated from pigs between 1994 and 2010 in Great Britain (GB) highlighted the predominance of both *S*. Typhimurium and *S*. Derby in the pig population; in addition, *S*. Typhimurium was the most reported serovar every year. Nevertheless, a decreasing trend in *S*. Typhimurium isolation was observed during this study, while at the same time, an increasing trend was registered for *S*. 1,4,[5],12:i:-, which accounted for 25% of all isolates in 2010, making it the second-most isolated serovar from pigs that year [62]. In the 2017–2021 time period, *S.* 1,4,[5],12:i:- and *S.* Typhimurium accounted, together, for more than 70% of all isolations from pigs in livestock productions in the United Kingdom [136].

With regard to the US, *S.* 1,4,[5],12:i:- was the most isolated serovar from swine in 2016, followed by *S*. Typhimurium and *S*. Derby (33%, 15%, and 9.6%, respectively), considering both clinical and non-clinical cases [137]. In addition, *S*. 1,4,[5],12:i:- has become the predominant serovar isolated from swine samples analyzed by veterinary diagnostic laboratories in the US since 2014 [64].

### 6.2. Salmonella Prevalence at Slaughterhouse

In a study comparing the on-farm prevalence of *Salmonella* infections in pigs to prevalence at slaughter, it was reported that on-farm prevalence often seemed to be lower, in part because of latent, undetectable carriers [138], which led to an underestimated on-farm prevalence [139]. Furthermore, *Salmonella* could be shed only after carrier animals had left the farm, mainly due to stress related to transport, commingling with different animals, and lairage [140], even after a short-term exposure [73,130]. In addition, the longer the time pigs spent in lairage, the higher the risk of *Salmonella* infection [141]. Lairage duration has been positively associated with *Salmonella* detection in pigs’ lymph nodes; it was reported that when the lairage time exceeded 12 hours, pigs were most likely to be infected with *Salmonella* from the lairage environment compared to pigs held in lairage for approximately 1–3 hours (16.7% versus 11.1%) [142].

Animals’ transport to the slaughterhouse, as well as transport time, together with stress induced by handling, feed withdrawal, and commingling, could exacerbate *Salmonella* shedding by infected animals, and simultaneously increase infection probability in healthy pigs [20,130,143]. In addition, both holding pens and trucks that are contaminated with *Salmonella* before and after transportation increase the possibility of infections [140,144].

At slaughter, pigs infected with *Salmonella* can carry the microorganism on the skin, in the oral cavity, in feces, and in lymph nodes [20,141]; this could potentially lead to carcass cross-contamination during slaughtering stages [129,145]. Furthermore, during slaughter, pig meat could be contaminated with *Salmonella* because of incorrect evisceration practices or when hygiene measures are not properly followed, which could involve the accidental leakage of cecal content, mainly feces. In turn, this could lead to carcass contamination [130,145,146]. A Dutch study estimated that approximately 55–90% of carcass contamination takes place during the evisceration process [145]. It has also been demonstrated that pigs coming from farms with a high *Salmonella* fecal positivity had higher rates of carcass contamination at slaughter [10], and that *Salmonella* isolation from ileocecal lymph nodes is believed to accurately reflect on-farm prevalence [73,130,147]. 

The 2006–2007 EFSA survey, which was conducted with the aim of evaluating *Salmonella* prevalence in slaughter pigs in the EU, reported a prevalence of 10.3% in lymph nodes (data from 24 MSs) and of 8.3% for carcass swabs (data from 13 MSs) [41]. This survey also ascertained a great deal of variability among different MSs [148]. Furthermore, it is worth pointing out that in the EU, there are still no harmonized programs for *Salmonella* control in the pig production chain, even though some MSs apply their own monitoring plans [13]. For example, Denmark, Estonia, Finland, Germany, Norway, and Sweden are applying *Salmonella* control programs in the swine population [149]. According to more recent data from European Competent Authorities (CAs), in the 2017–2021 period, the number of *Salmonella*-positive pig carcasses was lower (1.7–3.6%) than that reported in the EFSA survey for the 2006–2007 period. Data from food business operators’ (FBOp) self-monitoring were lower compared to data from CAs (Table 2). 

In 2019, an abattoir-based *Salmonella* prevalence study performed in GB found an overall prevalence of 32.2% from cecal samples, in which *S*. 1,4,[5],12:i:- was the most common serovar (36.6%); moreover, *S*. 1,4,[5],12:i:- and *S*. Typhimurium, together, accounted for 41.1% of all isolates, further highlighting the close relationship between these serovars and swine [151]. In addition to this, an Irish report, in which pooled lymph nodes and cecal material were analyzed, evidenced a high *Salmonella* prevalence at slaughter (pooled cecal content—55.5%, ileocecal lymph nodes—31.7%, carcass swabs—11.5%), with *S*. Typhimurium and *S*. 1,4,[5],12:i:- accounting for 65% of all the isolates. This is consistent with data reporting that these two serovars are among the most circulating ones in swine across Europe [147].

In the US, with regard to the *Salmonella* positivity rate in market hogs’ and sows’ cecal content at slaughter, data coming from USDA-FSIS evidenced a *Salmonella* positivity rate ranging from 34.7% to 49.7% in market hogs and from 50% to 67.9% in sows from 2013 to 2021; *S*. Derby, *S*. Typhimurium, and *S*. 1,4,[5],12:i:- were among the most frequently reported serovars [152,153].

Compared to the EU, other countries are characterized by a higher *Salmonella* prevalence in swine at slaughter [154]. In 2011, a Chinese study assessing *Salmonella* prevalence at pig slaughterhouses in three districts of Henan Province reported a prevalence of 29.2% (considering both carcass surface swabs and lymph nodes), in which *S*. Typhimurium was the most isolated serovar (28.6%), followed by *S*. Derby (27.1%) [155]. Later on, in 2016, an overall *Salmonella* prevalence of 22.9% was registered in slaughterhouses in Wuhan Province after the analysis of rectal swabs, carcass swabs, and pork samples [156].

### 6.3. Salmonella Prevalence in Pig Meat

In recent years, *S*. 1,4,[5],12:i:-, *S*. Typhimurium, and *S*. Derby have been the most reported serovars out of serotyped isolates from pig meat in the EU (Table 3) [133,134,135]. For example, *S*. 1,4,[5],12:i:-, *S*. Derby, and *S*. Typhimurium accounted for, respectively, 26.6%, 21.3%, and 14% of serotyped *Salmonella* isolated from pig meat in the EU in 2019 (Table 3), further underlying the tight connection between these three serovars and the pig production chain.

In the US, according to NARMS, in the 2013–2021 period, *Salmonella* prevalence in retail pork samples ranged from 0.8% to 4.5% [152].

According to a Chinese report evaluating *Salmonella* prevalence in food samples collected from 2011 to 2014, the vast majority of *S*. 1,4,[5],12:i:- isolates (84.6%) came from beef and pig meat products considered together [55].

To summarize, the main serovars responsible for infection in pigs in Europe, in the US, and in China are *S*. Typhimurium, *S*. 1,4,[5],12:i:-, and *S*. Derby. Available data highlight the existing correlation between these serovars and the pig production chain. Taken together, the collected information highlights the urgency of strengthening and improving *Salmonella* control measures worldwide, with the aim of reducing *Salmonella* prevalence in pigs and in pig-derived foodstuffs. 

## 7. *Salmonella* Serovars Associated with Human Infections and Correlation to Pork Products 

In the EU, in the years 2017–2021, *S*. Typhimurium and *S*. 1,4,[5],12:i:- were, respectively, the second- and the third-most isolated serovar from human salmonellosis cases, after *S*. Enteritidis, while *S*. Derby was the fifth-most isolated serovar from humans, albeit with a lower prevalence (Table 4) [13].

The most isolated serovars from pigs (*S*. Typhimurium, *S*. 1,4,[5],12:i:-, and *S*. Derby) are also among the most reported serovars isolated from human salmonellosis cases in the EU in recent years, although *S*. Derby is reported with a lower prevalence as opposed to *S*. Typhimurium and *S*. 1,4,[5],12:i:- (Table 4). In addition to these data, *S*. Typhimurium was the second main serovar involved in human outbreaks (9%), followed by *S*. 1,4,[5],12:i:- (1.6%) in 2018 in the EU [134]. *S*. Derby high prevalence in swine and relatively lower prevalence in humans could be explained by the lack of some virulence-associated genes [7]. Furthermore, in the EU, in 2020, pork products were the foodstuffs most involved in human salmonellosis outbreaks after eggs and egg-derived products. *S*. Typhimurium was and still is mainly related to poultry and pigs, while *S*. 1,4,[5],12:i:- and *S*. Derby are primarily linked to swine [150].

In the US, a total of 902 foodborne outbreaks were registered in 2015. *Salmonella* was the second-most prevalent cause of illness, being responsible for 34% of the outbreaks and for 39% of all the illnesses. Pork products were among the top three main food categories involved; *S*. Enteritidis was the most isolated serovar (35%), followed by *S*. 1,4,[5],12:i:- (10%) [157]. Since 2011, according to reports made by the laboratory-based Enteric Disease Surveillance system, the *S*. 1,4,[5],12:i:- human cases incidence rate has increased by 580% in the US. This considerable rise could be explained in part by the increased awareness toward this serovar [158]. In 2016, a total of 46,623 human salmonellosis cases were recorded, with both *S*. Typhimurium (9.8%) and *S*. 1,4,[5],12:i:- (4.7%) among the five most isolated serovars in the US (third and fifth, respectively) [158].

Moreover, the role played by pigs in the transmission of *Salmonella* to humans via contaminated food products has been documented, and several studies have highlighted that *S*. 1,4,[5],12:i:- and *S*. Typhimurium strains isolated from pork were responsible for foodborne outbreaks in humans (Table 5) [13,135,150].

Taking *S*. Choleraesuis into consideration, to the best of our knowledge, there is a paucity of information about foodborne outbreaks caused by this serovar, mainly because of its low incidence in humans, given that this serovar is swine-adapted. Nevertheless, in 2021, in Northern Italy, according to data from our institution, five *S*. Choleraesuis human cases showed a high degree of genomic similarity with one isolate coming from swine in the Emilia-Romagna region (unpublished data).

In conclusion, in order to control *Salmonella* along the pig production chain, the provided data strengthen the need to apply rigorous hygiene policies throughout the entire production system, coupled with surveillance systems implementing an interdisciplinary, holistic approach, in accordance with the One Health paradigm [159].

**Table 5:** Example of pork product-derived *Salmonella* foodborne outbreaks (2004–2021), associated with *S*. 1,4,[5],12:i:- and *S*. Typhimurium in the EU and in the US. Modified from Campos et al., 2019 [11].

Serovar	Year(s)	Country(ies)	Human Cases	Infection Source	Reference
	2006	Luxembourg	133	Pork meat	[160]
***S*** **. 1,4,[5],12:i:-**	2010	France	69	Dried pork sausage	[161]
	2011	Italy	16	Cooked pork product	[162]
	2011	France	337	Dried pork sausage	[163]
	2011	Spain	38	Dried pork sausage	[164]
	2013	Italy	NS	Pork salami	[165]
	2013	Germany	61	Minced pork	[166]
	2015	USA	188	Pork meat	[167]
	2017	Greece	37	Pork meat	[168]
	2018	England	15	Pork	[169]
	2018–2019	Denmark	49	Raw pork sausage	[170]
	2020–2021	France	11	Dried pork sausages	[171]
	2021	USA	34	Italian-style salami sticks	[172]
***S.* Typhimurium**	2004	Italy	63	Pork salami	[173]
	2005	Denmark	26	Pork products	[174]
	2008	Denmark, Norway, Sweden	37, 10, 4	Danish pork meat, minced meat	[175]
	2008	Denmark	1054	Pork products	[176]
	2010	Italy	30	Pork salami	[177]
	2010	Italy	5	Pork salami	[177]
	2010	Denmark	20	Pork salami	[178]
	2010	Denmark	172	Pork products	[179]
	2011	Denmark	22	Smoked pork tenderloin	[180]
	2011	England	51	Pork products	[181]
	2011	Spain	8	Dried pork sausage	[164]
	2018	England	28	Pork	[169]
	2021	USA	26	Italian-style meats	[182]

## 8. Surveillance of Foodborne Salmonellosis Outbreaks

Foodborne outbreaks due to *Salmonella* spp. have a strong impact on public health, causing illness with high hospitalization rates and significant socio-economic costs [183]. Taking this into consideration, laboratory-based surveillance is one of the pillars in the field of infectious disease monitoring [184]. Typically, it is carried out by public health and clinical laboratories. These laboratories collect and analyze samples from patients who are suspected to be ill with foodborne diseases. Then, positive cases are reported to public health authorities and strains are sent to reference laboratories for further testing [185,186]. Therefore, laboratory-based surveillance is a crucial part of monitoring foodborne diseases at a worldwide level, with the aim of collecting data on zoonoses trends and to detect and confirm outbreaks [185,187]. As previously stated, most salmonellosis cases in humans are associated with the consumption of contaminated animal-derived food products, such as pig meat, although in most cases, the origin of the infection is difficult to identify. The assignment of possible infection sources needs methods for targeted pathogen monitoring and rapid cluster detection, in order to reduce the burden posed by this disease and to limit the spread of infections [188].

Prior to the advent of whole genome sequencing (WGS), traditional bacterial typing techniques such as serotyping, phage typing, PFGE, MLVA, and MLST (multilocus sequence typing) were the standard typing tools for several foodborne bacterial pathogens [189]. Even though these techniques are still employed all over the world, they lack the ability to accurately discriminate between isolates during outbreaks [189,190]. In recent years, WGS has emerged as a powerful tool for genotyping pathogenic strains, including *Salmonella*, showing greater sensitivity and specificity compared to traditional methods [191,192]. WGS allows for an increased ability to discriminate reliably between isolates from the same species that are related, thus enhancing outbreak detection and discrimination power [193]. As a result, WGS is regarded as an “all-in-one” test, since data that would normally be obtained by a combination of traditional typing techniques can be retrieved in silico from sequencing data [194]. WGS has become and continues to become a key feature of public health surveillance systems [195,196]. Public health laboratories are implementing WGS approaches to better track and manage *Salmonella* foodborne-derived outbreaks [197] and, as of lately, many public health laboratories have successfully transitioned to an integrative WGS surveillance system of clinically relevant pathogens, such as PulseNet in the US [195]. To conclude, WGS has made it possible not only to perform better discrimination between outbreak-related and sporadic isolates but also to connect sporadic human cases to specific foodstuffs or animal sources; moreover, it allows us to identify contamination points across the food chain, which enables interventions during contaminated product trace-back analysis and eventual product recalls [189]. Nevertheless, it is important to acknowledge that to perform meaningful and accurate epidemiological surveillance, it is crucial to combine genomic evidence with clinical and epidemiological data [196]. Moreover, to prompt better inter-laboratory coordination and communication, standardized international protocols and workflows must be implemented [198,199], in order to improve and to guarantee microbial food safety and consumer protection. This translates to a transdisciplinary and multisectoral “One Health” approach, aimed at managing the threat posed by foodborne diseases, with the collaboration of experts comprising human, animal, and environmental health [200].

## 9. Conclusions

Salmonellosis and *Salmonella* infections in pigs are a problem for both animal and human health. Swine salmonellosis causes economic losses due to its lethality rate, growth retardation, and the need for antimicrobial usage. Salmonellosis also has an important impact on human health, as it is one of the most frequently reported foodborne diseases in EU countries. As reported, pork products are one of the main sources responsible for human salmonellosis. As of now, *S*. Typhimurium, *S*. 1,4,[5],12:i:-, and *S*. Derby are the most prevalent serovars linked to the pork production chain at a worldwide level. For this reason, it is important to improve knowledge about the role of carrier pigs, the epidemiology of the infection, the on-farm risk factors, the distribution of *Salmonella* serovars among pigs, and contamination routes at slaughter.

Different risk factors can influence *Salmonella* prevalence at the farm level, and its reduction needs different measures to be combined. Mitigation strategies should be adopted or reinforced, mainly regarding on-farm biosecurity, uncontaminated feed provision, quarantine of newly introduced animals, and implementation of cleaning and disinfecting practices. Vaccinating animals could contribute to decreasing *Salmonella* prevalence at slaughter, and procedures to prevent or minimize cross-contamination should be reinforced at the retail level. Measures at the farm level should be associated with strategies to reduce the risk of animal infection and carcass contamination in the stages immediately preceding slaughter. Transportation practices and holding at slaughter are crucial in influencing the prevalence of pigs positive for *Salmonella* entering the slaughter chain. In particular, proper hygienic practices showed a huge importance in reducing the level of contamination of carcasses at slaughter, reducing the persistence of *Salmonella* in the slaughter environment, and preventing the subsequent spread of *Salmonella* to pig carcasses.

Measures for reducing prevalence must be associated with effective *Salmonella* monitoring along the food chain, based on laboratory surveillance, in order to connect sporadic human cases to specific foodstuffs or animal sources; moreover, it allows us to identify contamination points across the food chain that enable interventions during contaminated product trace-back analysis and product recalls. Hence, more powerful control measures must be applied within an integrative and global “One Health” surveillance system, coordinating different stakeholders such as farmers, veterinarians working on animal health and food safety, and regulatory agencies.

## Figures and Tables

**Table 1:** Prevalence of *S*. 1,4,[5],12:i:- and of *S*. Typhimurium in serotyped *Salmonella* isolates from pigs in 2017–2019 and in 2021 in the EU.

Year	*S*. 1,4,[5],12:i:-	*S*. Typhimurium	Reference
2017	37.4%	20.6%	[133]
2018	25.7%	12.4%	[134]
2019	28.8%	12.7%	[135]
2021	28.2%	15.3%	[13]

Data not available for 2020.

**Table 2:** Pigs’ carcasses testing positive for *Salmonella* (before chilling and after dressing) in the EU from 2017 to 2021; data coming from CAs and FBOp.

Year	CAs	FBOp	Reference
2017	2.15%	1.85%	[133]
2018	2.69%	1.57%	[134]
2019	3.15%	1.51%	[135]
2020	3.60%	1.70%	[150]
2021	1.70%	1.40%	[13]

**Table 3:** Percentages of the most reported *Salmonella* serovars out of serotyped isolates from pig meat in the EU from 2017 to 2019.

Year	*S*. 1,4,[5],12:i:-	*S*. Typhimurium	*S*. Derby	Reference
2017	22.0%	27.0%	NS*	[133]
2018	12.4%	13.9%	20.6%	[134]
2019	26.6%	14.0%	21.3%	[135]

NS*, not specified.

**Table 4:** Salmonellosis-confirmed human cases in the EU per year from 2017 to 2021, together with the prevalence of *S*. Typhimurium, *S*. 1,4,[5],12:i:-, and *S*. Derby associated with human cases.

Year	Human Cases	*S*. Typhimurium	*S*. 1,4,[5],12:i:-	*S*. Derby	References
2017	91,662	13.4%	8.0%	0.8%	[133]
2018	91,858	13.0%	8.1%	0.9%	[134]
2019	87,923	11.9%	8.2%	0.9%	[135]
2020	52,702	12.4%	11.1%	1.2%	[150]
2021	60,050	11.4%	8.8%	0.9%	[13]

## Data Availability

No new data were created or analyzed in this study.
